# Through the lens of rural patients and pharmacies: A content analysis of state level pharmacy benefit manager regulations and policies

**DOI:** 10.1016/j.rcsop.2025.100595

**Published:** 2025-03-25

**Authors:** Tyler C. Melton, MaryKathleen Ryan, Andrew M. Stallings, Sang H. Park, Cameron Lanier, Jordan Marie Ballou, Meagen Rosenthal

**Affiliations:** a University of Tennessee Health Science Center College of Pharmacy, Department of Clinical Pharmacy and Translational Science, United States of America; b Ballad Health at Johnson City Medical Center, United States of America; c Clinical Pharmacy and Outcomes Sciences, University of South Carolina College of Pharmacy, United States of America; d School of Pharmacy at the University of Mississippi, United States of America

**Keywords:** Pharmacy benefit manager, Rural health, Rural pharmacy

## Abstract

**Background:**

Pharmacy Benefit Managers (PBMs) are responsible for establishing community pharmacy reimbursement practices and prices to varying degrees. Understanding PBMs' reimbursement practices is necessary for the continued viability of community pharmacies located in underserved and rural patient communities. Currently, there is a gap in literature exploring PBM reform and the impact this legislation has on rural pharmacy practice.

**Objectives:**

This content analysis reviews the legislation complied by the National Community Pharmacists Association (NCPA) and determines its benefits to pharmacies and patients in rural areas.

**Methods:**

The NCPA PBM Reform legislation document included bills from 48 states and the District of Columbia, that were introduced between November 30, 2018, through December 7, 2021. Bills were classified as enacted (*n* = 81), in debate (*n* = 186), or as having failed to be enacted (*n* = 120). Eighty-one enacted bills were reviewed to assess if it benefited patients, pharmacies, or both. Bills not benefiting either pharmacies or patients were excluded.

**Results:**

Fifty-seven bills were included in the content analysis, where six categories were identified using thematic analysis and classified as: PBM Operations, Drug Pricing, Transparency, Reimbursements, Cost Sharing, and Prior Authorization. Only twenty-two bills were identified as potentially benefitting both rural pharmacies and rural patients through inclusion of legislation managing PBM practices involving patient steering, network adequacy, pricing transparency, reforming cost-sharing structures, and streamlining prior authorization processes.

**Conclusions:**

This study identifies multiple PBM legislation categories having the potential to impact rural pharmacy operations and patient outcomes. However, further research is needed to understand the specific financial and clinical impact of these PBM legislation categories on rural communities and rural pharmacy practice, as well as their alignment with enabling pharmacists to combat unique health disparities and challenges facing rural communities.

## 1 Introduction

Pharmacy Benefit Managers (PBMs) are third-party organizations acting as intermediaries between insurance companies, pharmacies, and drug manufacturers. Currently, PBMs are highly integrated into the healthcare system through their various roles, including developing drug formularies and negotiating drug prices and rebates with manufacturers.^1^ They also perform administrative functions on behalf of insurance companies and handle pharmacy reimbursements.^1^

The reimbursement process consists of pharmacies stocking and dispensing medications while PBMs are responsible for managing copays and determining the amount to reimburse a pharmacy through negotiated contracts.^2^ However, PBMs have proprietary practices and methodologies that make it unclear how values for reimbursement are determined.^3^ For example, one pricing model incorporates the Maximum Allowable Cost (MAC) list that is meant to keep healthcare costs low by encouraging pharmacies to obtain medications at a low cost, promoting competition between manufacturers, and increasing dispensation of generic medications.^4^ In practice however, pharmacies may operate at a loss due to being reimbursed by PBMs for a lower MAC amount than the cost to purchase and dispense the medication when a different pricing model or cost is used.^2^^,^^3^ Without relief, pharmacies operating at a sustained loss face uncertain survival, which may contribute to the rise in pharmacy closures over the past 20 years.^1^^,^^5^ Continued closure of independent pharmacies is a particular concern for rural communities which often rely on these establishments as their only source of medications and local healthcare resources.

Additional problematic PBM practices include gag clauses, patient steering, and transparency or reporting practices. Gag clauses are provisions in contracts between pharmacies and PBMs or insurers that prevent pharmacists from voluntarily disclosing to patients when they could pay less for a prescription by not using their insurance (e.g., paying the cash price), thereby decreasing medication affordability.^6^^,^^7^ Continued legislative efforts have been made federally and at state levels to ban these practices. Patient steering involves guiding patients towards specific types of pharmacies, such as corporate-owned retail, mail-order, or specialty pharmacies, often as part of cost management strategies. Rural independent pharmacies, typically operating with a smaller customer base, face substantial business losses due to this redirection of prescriptions.^8^ This threatens the financial sustainability of these vital rural pharmacies and diminishes competition, potentially leading to higher drug prices and limited pharmaceutical options for the community.^9^ Improved transparency, conveyed in plain language instead of technical jargon, can increase health literacy and enable patients to make informed decisions about their medication costs.^10^ Such clarity is especially vital in rural areas, where limited healthcare options can make effective budgeting a significant aspect of household finances.

Additionally, legislation preventing PBMs from barring pharmacists from sharing information about cheaper medication alternatives fosters a collaborative environment between patients and pharmacists, which is essential for managing healthcare costs effectively.^6^ Network adequacy in rural communities is of paramount importance as these oftentimes isolated or difficult to reach communities may face limited pharmacy options within convenient distances.^11^ For example, in West Virginia, PBMs are required to contract with a mix of both mail-order and retail pharmacies (WV HB 2263). Other states have required PBMs to ensure a network pharmacy is within 15 miles of at least 70 % of people living in rural areas (AR HB 1804). Additionally, other states have recognized the importance of network adequacy by prohibiting Medicaid services from contracting with a managed care organization that uses a PBM demonstrating unfair practices towards pharmacies with fewer than 7 locations (MI SB 82).

The trend of rural pharmacy closures, often attributed to decreased local patronage, has implications that extend beyond immediate healthcare concerns.^5^ The dynamics of patient steering by PBMs, and health plans are essential factors to consider in the context of rural pharmacy closures. These practices, often aligned with the business objectives of PBMs and health plans, may coincide with challenges rural communities face, including economic stability and access to healthcare.^12^ Recognizing these potential correlations, it becomes evident that a thoughtful examination of these practices on rural health could be beneficial.

Advocacy for pharmacies is especially important in rural areas to prevent closures and additional barriers to care for patients living in rural spaces. The National Community Pharmacists Association (NCPA) is an organization that advocates for independent pharmacies. In an effort to inform advocacy efforts, the NCPA maintains a list of legislation pertaining to PBM reform in various phases of the legislative process.^13^ There is currently a gap in pharmacy literature concerning the impact PBM legislation has on rural pharmacy practice and communities. This study aims to review the legislation compiled by the NCPA to determine how this oversight benefits pharmacies and patients in rural communities.

## 2 Methods

**Fig. 1:**
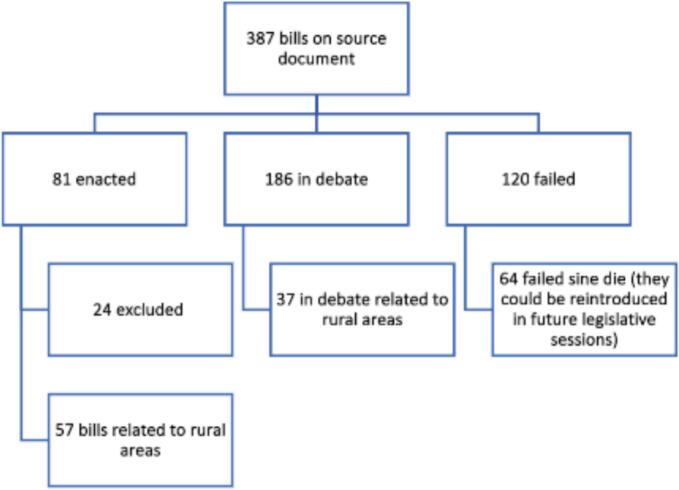
Selection process.

The bills analyzed in this study were sourced from December 9th^,^ 2021, update of the National Community Pharmacists Association (NCPA) PBM Reform legislation document.^13^ This update contained 397 bills, from 48 states and the District of Columbia, that were introduced between November 30th, 2018, through December 7th, 2021. These bills were classified by the research team as enacted (*n* = 81), in debate (*n* = 186), or as having failed to be enacted (*n* = 120) (Fig. 1). Enacted bills were further analyzed to assess if the legislation benefited patients, pharmacies, or both. The classification of these benefits were decided by two reviewers and a pharmacist practicing in rural community pharmacy. Benefits to patients could include cost-savings, accessibility to healthcare, improvement in health outcomes, access to timely care, and autonomy to choose desired pharmacies. Benefits to pharmacies included profit margins, positive impact to pharmacy practice, and providing effective and timely pharmaceutical care. Bills were excluded from analysis if they did not pertain to rural patients, rural pharmacy practice, or disparities facing rural communities. For the purposes of this content analysis rural was defined using the Federal Office of Rural health Policy (FORHP) criteria including geographic and demographic considerations^14^.

If the NCPA document lacked the necessary description to understand an enacted bill, the bill was searched for on the corresponding state legislature's website and assessed using key terms including: PBM, pharmacy benefit manager, and/or pharmacy. The bill was then reviewed and analyzed. Legislations were labeled as being of benefit to pharmacy, patient, or both through independent assessment by two reviewers, with disagreements being resolved by a third reviewer. Final analysis and categorization of the legislation was conducted by three reviewers, consisting of one pharmacist and two pharmacy student research assistants. Legislation was deductively categorized based on the bill's focus including: PBM Operations, Drug Pricing, Transparency, Reimbursements, Cost Sharing, and Prior Authorization. Discrepancies in categorization were addressed collectively by the three reviewers and resolved using open discussion. No mediation was necessary for discrepancy resolution. Established categories were discussed, adjusted, and finalized based on consensus from the research team.

## 3 Results

Of the 81 enacted bills from the NCPA December 2021 PBM Reform legislation document, 24 bills were excluded from the content analysis as they did not relate to rural areas. Fifty-seven bills were determined to have potential benefits for rural patients and/or rural pharmacy practice and are explored in further detail hereafter (Fig. 1). This content analysis identified 6 legislative categories, including: PBM Operations, Drug Pricing, Transparency, Reimbursements, Cost Sharing, and Prior Authorization (Table 1). These six categories were not uniformly represented across all states (Table 2). The categories with the highest representation were PBM Operations, Drug Pricing, and Transparency.

**Table 1:** Categorization of PBM Reform Legislation.^11^, ^15^, ^16^, ^17^, ^18^, ^19^, ^20^

Category	Description
PBM Operations	Regulation focused primarily on registration and protecting patient choice topics including: -Licensure and registration -Patient steering/freedom of choice -“Gag” clauses -Penalties for mailing and delivery services
Drug Pricing	Regulation focused on drug price limitations and frequent updates to prices topics including: -MAC pricing updates -Price caps -340B drug pricing nondiscrimination -Price hikes on generics
Transparency	Regulation focused on transparency and reporting topics including: -Current Wholesale Acquisition Costs (WAC) -Cost, benefit, and coverage data to covered entities
Reimbursements	Regulation focused on pharmacy reimbursement topics including: -Reimbursements to pharmacy not lower than costs -Reporting costs to patients -Reimbursement rate differences between pharmacies
Cost sharing	Regulation focused primarily on costing sharing topics including: -Copay accumulator program
Prior Authorization	Regulation focused primarily on prior authorization topics including: -Step therapy elimination for specific instances -Uniform prior authorization process

**Table 2:** NCPA PBM Reform Enacted State Specific Legislation.^15^

PBM Reform Category	Number of States with Legislation	Enacted
PBM Operations	18	AL, AR, CO, IL, KY, LA, MD, ME, MI, MT, NC, NH, OK, TN, TX, VA, WI, WV
Drug Pricing	17	AL, CO, IL, IN, NC, ND, OK, TN, UT, VT, WV
Transparency	15	CO, DE, IL, MD, ME, MT, ND, NV, OK, SD, TN, TX, WI, WV
Reimbursements	7	AL, DE, MD, RI, TN, TX, WV
Cost Sharing	4	AR, CT, LA, OK
Prior Authorization	4	AR, AZ, CA, NV

AL: Alabama, AR, AR: Arkansas, AZ: Arizona, CA: California, CO: Colorado, CT: Connecticut, DE: Delaware, IL: Illinois, IN: Indiana, LA: Louisiana, ME: Maine, MD: Maryland, MT: Montana, Nevada: NV, NC: North Carolina, ND: North Dakota, OK: Oklahoma, RI: Rhode Island, SD: South Dakota, TN: Tennessee, TX: Texas, UT: Utah, VT: Vermont, WV: West Virgina, WI: Wisconsin.

### 3.1 Category: PBM operations

The PBM Operations category consisted of three themes, including PBM licensure or registration, patient steering, and network adequacy. PBM licensure or registration was the most frequently occurring category with 8 of the 18 states passing laws pertaining to PBM Operations specifically requiring PBM licensure or registration. The themes with the greatest applicability to both patients and pharmacies included the prohibition of mail-order-only pharmacy services and a practice referred to as “patient steering”, wherein patients are incentivized and persuaded to use in-network or affiliated mail order-only pharmacies instead of the pharmacy the patient used in the past.^16^ Network adequacy within this category relates to a PBM's ability to provide reasonable access to needed care without unreasonable delay.^11^

### 3.2 Category: transparency

Transparency was the second most common category and refers to the degree of openness and clarity of business practices concerning various entities. Nine states have enacted legislation requiring quarterly or annual reporting of rebates, pricing, current WAC and justification for increasing drug prices from some or all the following entities including: PBMs, manufacturers, wholesalers, and managed care organizations (MCO).^15^ As some of this information is confidential in nature, only a portion, if any, is currently disclosed to the public. The main measure to increase transparency requires PBMs to provide cost, benefit, and coverage data to contracted entities and patients when requested.^17^

### 3.3 Category: drug pricing & reimbursement

Drug Pricing reform laws were enacted in 11 states and focused on updating MAC pricing practices and highlighted nondiscrimination towards 340B pharmacies. MAC pricing, as a payment system, is established through mutual agreement among market participants. This model guarantees that purchasers of health insurance, encompassing individual consumers, are protected from excessive costs for generic medications.^18^ Nondiscrimination included prohibiting practices such as exclusion of 340B pharmacies from the network, reimbursing 340B pharmacies at lower rates, and requiring pharmacies to disclose 340B status to PBMs. Moreover, reimbursement reform bills were the 4th most identified category and were enacted in 7 states. In Alabama, Delaware, Maryland, and Texas PBMs are no longer allowed to reimburse one pharmacy at a rate lower than they would reimburse themselves or an affiliate.^13^ Secondly, Tennessee and West Virginia implemented laws prohibiting PBMs reimbursing an amount less than the cost of obtaining the medications. These laws are designed to foster an equitable and cost-effective drug pricing system by eliminating unfair pricing disparities and ensuring pharmacies are reimbursed equally for identical services. This uniform PBM standard aims to prevent discriminatory reimbursement rates among pharmacies and ultimately help community pharmacies avoid operating at a profit loss.

### 3.4 Category: prior authorization & cost sharing

Prior Authorization and Cost Sharing categories appeared least often compared to others. Prior authorizations (PA) and step therapy are strategies used by MCOs and PBMs to decrease costs and ensure patients/providers follow guideline recommendations for therapy selection and escalation.^19^ These strategies decrease unnecessary use of expensive medications by ensuring patients first utilize less expensive medication choices or have clinical reason(s) preventing the use of other, less costly, alternatives. Furthermore, Cost-Sharing reform bills focused primarily on prohibiting copay accumulator programs, which prevents manufacturer coupons from counting towards a patient's deductible or out-of-pocket contribution.^20^ In Oklahoma, prohibiting these programs requires insurance companies to include all payments on behalf of the patient towards their out-of-pocket contribution (OK HB 2678). Whereas in other states, only the copayments and coinsurance contribute to the patient's out-of-pocket contributions (TX HB 2090). When patients decide to pay retail cash price for prescriptions, the payment does not count towards out-of-pocket requirements.

## 4 Discussion

Understanding the unique needs of rural pharmacy practice and the factors influencing pharmacy service provision is critical for guiding effective PBM reforms yet remains an understudied area. The higher prevalence of pharmacy deserts—regions lacking retail pharmacies—in rural areas highlights the need to examine their causes and implications.^21^ Identifying and categorizing commonalities in state legislation is essential for understanding PBM reforms and shared legislative trends nationwide. This content analysis focused on PBM legislative measures affecting rural patients and pharmacies.

Rural patients and community pharmacies face unique challenges and disparities, often intensified by PBM policies and procedures. Addressing PBM reform through the lens of its benefits for rural pharmacies and patients provides a valuable perspective on the legislation's potential impact. While states like Vermont, Texas, and North Carolina have significant rural populations, it remains unclear whether rurality influences PBM reform activity levels.^22^ Although rural pharmacy practices are present nationwide, research on state-specific PBM reform needs is limited.

Nationally, the initial focus of PBM reform targeted transparency, regulation of anti-competitive practices, promotion of patient access, and the elimination of gag clauses. While state and federal legislation have begun addressing these priorities, additional regulation is needed to address issues such as reimbursement, cost-sharing, and prior authorization practices.^12^, ^23^ Arkansas, a largely rural state, was one of the first states to actively engage in legislative action against PBMs to preserve and maintain the viability of pharmacies in their state. In 2015, the Arkansas legislature passed Act 900 to prevent PBMs from reimbursing less than the cost of acquiring and dispensing the medication to help keep rural pharmacies open.^24^ The Pharmaceutical Care Management Association (PCMA), on behalf of the 11 largest PBMs, filed a lawsuit claiming that the Employee Retirement Income Security Act of 1974 (ERISA) preempted state law as it pertained to employee benefit plans. *Rutledge v PCMA* concluded with the Supreme Court ruling unanimously in favor of the Arkansas PBM reform. The Supreme Court ruled that the broad interpretation of ERISA used by PBMs to preempt Arkansas law did not apply.^25^ This decision provided greater support for state-level healthcare cost control efforts in Arkansas, and it has the potential to do the same for other states as well. Decisions like the one in Arkansas and others around the country have created an opportunity for greater political advocacy by the profession of pharmacy.

The elimination of “Gag Clauses” has been a prioritization of recent federal and state legislation. In 2018, Federal legislation introduced the “Patient Right To Know Drug Prices Act”, effectively prohibiting PBMs from introducing gag clauses in pharmacy contracts.^26^ Later, states such as Tennessee and South Dakota passed laws preventing PBM's ability to prohibit pharmacists from informing patients of lower cash prices and substituting expensive medications for cheaper equivalent alternatives (TN HB 1530, SD HB 1263). These legislative efforts not only aids in patient savings but also ensures the viability of rural pharmacies, allowing these pharmacies to continue offering diverse and essential health services to their communities. Additionally, legislation focusing on network adequacy and requiring PBMs to include rural pharmacies in their networks provides patients the opportunity to fill prescriptions for acute needs and not be reliant on otherwise unavailable delivery services, especially in areas with geographic barrier considerations and containing poor healthcare infrastructure.

MAC pricing has been a large concern for rural pharmacies and has previously been cited as a primary concern for overall sustainability.^27^^,^^28^ Although the formula for MAC pricing was not affected, the requirement for frequent updates may help rural pharmacies receive adequate reimbursement as fees fluctuate.^27^ The nondiscrimination laws for 340B pharmacies could help critical access hospitals and federally qualified health centers in rural areas continue to use the money saved through purchasing 340B medications to provide additional services for their surrounding communities.^29^ These services are essential for improving access to healthcare and maintaining community health.^29^

Legislative efforts to reform cost-sharing practices have focused on curbing the use of copay accumulator programs. Such programs traditionally did not count manufacturer coupons towards a patient's deductible or out-of-pocket maximum.^30^ This practice often resulted in higher patient costs once the coupons were exhausted, as they would then face unexpected out-of-pocket expenses. By mitigating the financial burden on patients through inclusive counting of all payments towards out-of-pocket costs, these reforms could increase access to medications for rural patients. This is crucial in areas where healthcare resources are already limited. Furthermore, these changes could also support the viability of independent pharmacies by increasing the volume of prescriptions they fill, as patients might be less inclined to forgo medication due to cost concerns.^31^^,^^32^

Lastly, legislation affecting prior authorizations (PA) can benefit patients in rural areas by decreasing wait times for claim approval or denial. As described by Popatia and colleagues, the PA process often requires a multi-step approach for claim adjudication.^33^ These prolonged wait times can delay access to timely care, exacerbate disease states, reduce adherence, and lead to adverse or life-threatening health outcomes.^34^, ^35^, ^36^ To address these challenges, states have enacted laws automatically approving PA requests if a decision is not made within a specified timeframe. Additionally, new laws have focused on limiting step therapy requirements in specific circumstances, such as metastatic cancer, where bypassing step therapy enables patients to receive necessary care more promptly (AR SB 99, CA AB 347, NV SB 290). For rural pharmacies, these reforms are critical, ensuring equitable and efficient healthcare access. By minimizing delays and removing unnecessary barriers, these laws prioritize timely treatment for patients, regardless of their geographic location.

### 4.1 Limitations

This study is not without limitations. First, the document used to find the laws represented a snapshot in time from November 2018 to December 7th, 2021. Although this may not be an all-inclusive list, this analysis examined PBM reformation laws from a new perspective through the lens of the impact on rural pharmacies and rural communities. Additionally, some bills on the source document were in debate when it was published online and may have later been enacted or failed. This study was conducted from the pharmacy perspective and completed by individuals with pharmacy practice experience with prior knowledge of PBM operations. This knowledge may have changed the way PBM Reformation Legislation was categorized despite measures to mitigate biases. To mitigate selection bias, the authors ensured that reviewers reached consensus on the inclusion and categorization of legislation.

## 5 Conclusion

This project highlights legislative efforts intended to mitigate the negative impacts of PBMs from the perspective of rural patients and community pharmacies. This project identified 22 bills that could have a potentially beneficial impact in rural spaces including limiting patient steering, ensuring network adequacy, increasing pricing transparency, reforming cost-sharing structures, and streamlining prior authorization processes. Achieving sustainability and efficacy in rural pharmacies demands a comprehensive approach, encompassing policy changes, financial support mechanisms, and an understanding of the specific healthcare needs of rural populations. Continued advocacy for research and policy reform is crucial to increase support for rural pharmacies. This endeavor is essential to mitigate healthcare disparities in rural areas, enhance healthcare access and quality, and ensure equitable access to healthcare for all individuals, regardless of their location.

## Funding support

No external funding was received to support writing of this manuscript.

## CRediT authorship contribution statement

**Tyler C. Melton:** Writing – review & editing, Writing – original draft, Visualization, Validation, Resources, Project administration, Methodology, Investigation, Formal analysis, Data curation, Conceptualization. **MaryKathleen Ryan:** Writing – review & editing, Writing – original draft, Visualization, Validation, Investigation, Formal analysis, Data curation, Conceptualization. **Andrew M. Stallings:** Writing – review & editing, Writing – original draft, Visualization, Investigation, Formal analysis. **Sang H. Park:** Writing – review & editing, Writing – original draft, Visualization, Validation, Investigation, Formal analysis, Data curation, Conceptualization. **Cameron Lanier:** Writing – review & editing, Writing – original draft, Visualization, Investigation, Formal analysis. **Jordan Marie Ballou:** Writing – review & editing, Writing – original draft, Visualization, Validation, Investigation, Formal analysis, Data curation, Conceptualization. **Meagen Rosenthal:** Writing – review & editing, Writing – original draft, Visualization, Validation, Methodology, Investigation, Formal analysis, Data curation, Conceptualization.

## Declaration of competing interest

The authors declare no conflicts of interest.
